# PRN: a preprint service for catalyzing R-fMRI and neuroscience related studies

**DOI:** 10.12688/f1000research.5951.2

**Published:** 2015-08-19

**Authors:** Chao-gan Yan, Qingyang Li, Lei Gao

**Affiliations:** 1 The Nathan Kline Institute for Psychiatric Research, Orangeburg, NY, USA; 2 Institute of Psychology, Chinese Academy of Sciences, 16 Lincui Rd, Chaoyang District, Beijing, 100101, China; 3 Department of Child and Adolescent Psychiatry, New York University Langone Medical Center, New York, NY, USA; 4 Editorial Office of PRN, the R-fMRI Network, Inc., New York, NY, USA; 5 Department of Radiology, the First Affiliated Hospital of Nanchang University, Nanchang, China

**Keywords:** Free-submission, Neuroscience, Open-access, “Peer viewed”, Preprint-hosting, R-fMRI

## Abstract

Sharing drafts of scientific manuscripts on preprint hosting services for early exposure and pre-publication feedback is a well-accepted practice in fields such as physics, astronomy, or mathematics. The field of neuroscience, however, has yet to adopt the preprint model. A reason for this reluctance might partly be the lack of central preprint services for the field of neuroscience. To address this issue, we announce the launch of Preprints of the R-fMRI Network (PRN), a community funded preprint hosting service. PRN provides free-submission and free hosting of manuscripts for resting state functional magnetic resonance imaging (R-fMRI) and neuroscience related studies. Submitted articles are openly discussed and receive feedback from readers and a panel of invited consultants from the R-fMRI Network. All manuscripts and feedback are freely accessible online with citable permanent URL for open-access. The goal of PRN is to supplement the peer reviewed journal publication system – by more rapidly communicating the latest research achievements throughout the world. We hope PRN would help the field to embrace the preprint model and thus further accelerate R-fMRI and neuroscience related studies, eventually enhancing human mental health.

## Introduction

Before submitting manuscripts to traditional scientific journals for peer review and publication, researchers in some fields routinely distribute the manuscripts as preprints within their fields. In this way, they receive early feedback, which may help in preparing articles for definitive submission as well as rapidly propagating novel ideas to their fields. The well-known central repository for preprints, arXiv ( http://arXiv.org), was founded in 1991 by Dr. Paul Ginsparg for the field of physics. It gradually expanded to include astronomy, mathematics, computer science, nonlinear science, quantitative biology, and statistics as scientists in these fields began to embrace preprints ( Ginsparg, 2011). arXiv now hosts close to one million fulltext preprints (983,739 as of November 1, 2014). Registered users of arXiv can submit manuscripts (multiple versions are allowed) and all users can freely browse, view and cite any articles. Although arXiv lacks rating systems or a feedback mechanism to let users recommend papers of interest to peers or to provide feedback to authors, it is still an invaluable resource for the fields it serves.

However, researchers’ attitude toward preprints varies depending on the field. The field of neuroscience has yet to adopt the practice of releasing preprints. Instead, neuroscientists commonly circulate their manuscripts to collaborators and colleagues for feedback before submission, but distribution is private and limited to small groups. The reason for such limited sharing might partly be the lack of central preprint services for the field. Only in 2013 did two preprint services dedicated to biology emerge for the field of life science ( Callaway, 2013; Van Noorden, 2012). The two preprint services, PeerJ Preprints ( https://peerj.com/preprints/) started by PeerJ, Inc. and bioRxiv ( http://biorxiv.org) launched by Cold Spring Harbor Laboratory, are providing preprint hosting services with online feedback and comment systems. It is expected that early feedback would be helpful for authors in revising and improving their articles for later peer review process of traditional journals. Furthermore, commenters can be acknowledged for their contributions in later publication. However, it is only the dawn of neuroscience preprints -- bioRxiv and PeerJ Preprints have only received 56 and 38 neuroscience papers, respectively (as of 11/1/2014, see Table 1). More efforts to facilitate adoption of the preprint model appear to be needed.

**Table 1.** Overview of neuroscience related preprint manuscripts on online preprint services (as of 11/1/2014).

Name	SCOPE	Initial	Link	Fulltext hosted	Neuroscience related	fMRI related
arXiv	Mathematics, physics, astronomy, computer science, quantitative biology, statistics, and quantitative finance.	August 14, 1991	arXiv.org	984,747	475*	142***
BioRxiv	All aspects of research in the life sciences but does not accept clinical studies or clinical trials.	November 11, 2013	biorxiv.org	825	56**	6***
PeerJ PrePrints	Biological Sciences, Medical Sciences, and Health Sciences	April 3, 2013	peerj.com/preprints	581	38**	5***

*: Number of articles returned by searching the key word “neuroscience” on arxiv.org. **: Number of articles in the neuroscience sub-category of the corresponding websites. ***: Number of articles returned by searching the key word “fMRI” on corresponding websites.

**Figure 1.**
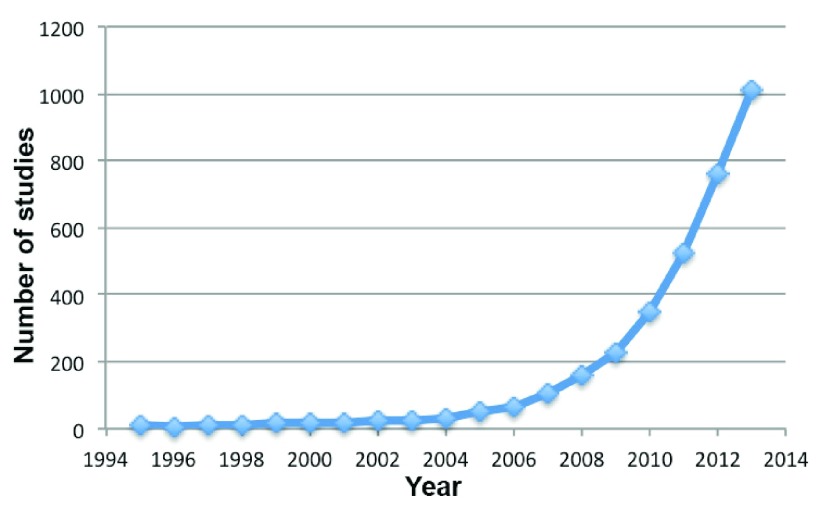
Number of R-fMRI related studies in PubMed (key words: “resting+state+fmri”).

A subfield of neuroscience, neuroimaging, especially that which focuses on resting-state functional magnetic resonance imaging (R-fMRI), has emerged as field which is embracing innovations such as open data sharing (e.g., ADHD-200-Consortium, 2012; Biswal *et al.*, 2010; Di Martino *et al.*, 2014; Hall *et al.*, 2012; Mennes *et al.*, 2013; Milham, 2012; Mueller *et al.*, 2005; Satterthwaite *et al.*, 2014; Van Essen *et al.*, 2013; Zuo *et al.*, 2014), open software sharing (e.g., Bellec *et al.*, 2012; Chao-Gan & Yu-Feng, 2010; Rubinov & Sporns, 2010; Sikka *et al.*, 2014; Song *et al.*, 2011; Taylor & Saad, 2013; Whitfield-Gabrieli & Nieto-Castanon, 2012; Xia *et al.*, 2013; Zang *et al.*, 2012; Zuo & Xing, 2014) and sharing of learning resources (e.g., Training Course in fMRI ( http://sitemaker.umich.edu/fmri.training.course) and The R-fMRI Course ( http://rfmri.org/Course)). As a method to investigate ongoing brain activity in basic, translational and clinical neuroscience research, R-fMRI has become an increasingly prevalent research area especially in recent years ( Fornito & Bullmore, 2012; Fox & Raichle, 2007; Kelly *et al.*, 2012; Van Dijk *et al.*, 2010) considering its sensitivity to characterize developmental, aging and pathological features ( Andrews-Hanna *et al.*, 2007; Fair *et al.*, 2008; Greicius, 2008; Zuo *et al.*, 2010), subject-friendly data collection procedures in clinical samples, and high comparability and consistency across studies and sites ( ADHD-200-Consortium, 2012; Biswal *et al.*, 2010; Mennes *et al.*, 2013; Tomasi & Volkow, 2012). This field has expanded exponentially, now exceeding more than 1000 studies published per year ( Figure 1). Given the emerging traditions of openness in this field, and an increasing number of researchers involved, we believe that the field can benefit from a preprint service that provides open viewing and commenting.

Accordingly, we are announcing a preprint publication model for catalyzing R-fMRI and related neuroscience studies. We have designed PRN as a community funded, open-access, free-submission, open-discussion, preprint service. The goal of PRN is to supplement the peer reviewed journal publication system by supporting more rapid communication of the latest research observations throughout the world.

## Implementation

We have implemented the PRN service based on the success of The R-fMRI Network (RFMRI.ORG). The R-fMRI Network (RFMRI.ORG) has been designed as a framework to support R-fMRI studies. The R-fMRI Network comprises R-fMRI researchers (the nodes) who are connected by sharing (the edges) with each other. Through the network, imagers can efficiently share ideas, comments, resources, tools, experiences, data, and increasing knowledge of the brain. Researchers (nodes) with basic neuroscience, methodological, or clinical backgrounds can connect with each other in the network. The R-fMRI Network currently has more than 5000 registered members, aiming to enhance collaborations among researchers, especially to translate our knowledge of basic neuroscience and methodology to clinical applications (bench to bedside).

The R-fMRI Network (RFMRI.ORG) is designed with a forum system and an integrated mailing list based on drupal ( http://drupal.org) and mailman ( http://www.gnu.org/software/mailman/). The source codes of the website are openly shared through Github ( https://github.com/Chaogan-Yan/rfmri.org). As an online forum system, The R-fMRI Network allows researchers to propose research ideas, discuss controversial issues, request help in using software, share experiences, report preliminary results, initiate collaborations and even seek jobs. The R-fMRI Network hosts several instances of R-fMRI software (e.g., DPABI, DPARSF and GraphVar), online learning resources, open data links, and gathers the latest R-fMRI related studies from PubMed. All new posts are sent to all R-fMRI Network registered users via an integrated mailing list, and users can comment on any post by directly replying to the mailing list.

The PRN has been built based on the existing infrastructure of RFMRI.ORG. Submission of a manuscript is as easy as posting a forum post with the paper title as the post title, manuscript title page and abstract as the post content and a PDF version of the fulltext manuscript as an attachment of the post. The preprint manuscript has a permanent online URL with a convenient commenting system as in the forum system, and with mailing list immediate notification to all registered users. Furthermore, PRN has been empowered with the following features.

## Features

### Preprint

All submissions to PRN are preprint submissions, thus authors can freely revise and submit unrevised or revised manuscripts to formal peer reviewed traditional journals which allow preprints. PRN only checks the format of manuscripts, and contacts the corresponding author to confirm his/her approval of submission. As a preprint service, PRN has no peer review process and no editing service.

### Open-access

All PRN articles are freely available online after submission. Readers can freely read, download and comment on articles. Like other posts at the R-fMRI Network, all submissions are dated, citable with a permanent URL and indexed by Google. Furthermore, each PRN submission has a unique URL with a time stamp such as http://rfmri.org/PRN_140828001.

The PRN does not ask the copyright of the work to be transferred, however, the PRN requires sufficient rights to distribute submitted articles in perpetuity as documented at http://rfmri.org/PRN_140831001. In general, the authors should grant the PRN a non-exclusive and irrevocable license to distribute the article, or certify the work is either under Creative Commons Attribution license, or the Creative Commons Attribution-Noncommercial-ShareAlike license.

### Free-submission

Unlike other open-access journals, submission to PRN is free of charge.

### Open-Discussion

Articles at PRN are openly discussed by interested readers and also by consultants. The PRN has enrolled a panel of consultants – each obligated to comment on three PRN papers per six-month period. On a monthly basis, PRN rates “consultants’ choice” and “readers’ choice” articles. Furthermore, PRN rates the most active articles, i.e., those elicited the most comments and revisions – as a way to spur feedback and revision of articles.

### Community funded

The PRN is a community funded effort. We encourage all researchers to make a small contribution at http://rfmri.org/HelpUs to help the PRN effort, but this is completely voluntary.

## Compatibility with traditional formal journals

A major concern is that traditional formal journals may refuse to publish manuscripts which have been previously made available online on a preprint server. To address this concern, a cross-field discussion on preprints has been initiated with editors-in-chief of journals in neuroscience, physics and mathematics. An editor-in-chief in physics responded that arXiv is invaluable for doing research in physics, and is scanned by most physicists every day. Several editors-in-chief of Neuroscience journals have confirmed that their journals do accept preprint manuscripts. Based on the information of Sherpa-Romeo ( http://www.sherpa.ac.uk/romeo), we have organized a table of PRN compatible journals ( http://rfmri.org/PRN_20140921001). The authors should pay close attention to the table ( http://rfmri.org/PRN_20140921001) before submitting preprint manuscripts to PRN, to avoid jeopardizing their subsequent submission to PRN-incompatible journals. The authors should reveal that the papers have been archived in PRN when submitting to preprint compatible journals. The comments and revisions are encouraged to be included in their submission.

## Conclusions

We have launched PRN as a preprint service for catalyzing R-fMRI and related neuroscience studies. By empowering this preprint system with an online commenting system and mailing list notification system to promote the newest studies to the R-fMRI community, as well as inviting R-fMRI experts as consultants to comment on preprint manuscripts, we hope PRN would help the field embrace the preprint model and thus accelerate R-fMRI and neuroscience related studies, eventually enhancing human mental health.
