# Supplementary material for: PRN: a preprint service for catalyzing R-fMRI and neuroscience related studies
Source: F1000Res. 2015 Aug 19;3:313. Originally published 2014 Dec 22. [Version 2] doi: 10.12688/f1000research.5951.2 (PMC4367516; doi:10.12688/f1000research.5951.2)
Supplement: Peer review history

# Peer review: Referee response for version 2

**DOI:** 10.5256/f1000research.7450.r10018

**Published:** 2015-09-08

**Authors:** Ze Wang

**Affiliations:** 1 Department of Psychiatry, University of Pennsylvania, Philadelphia, PA, USA

My comments have been fully addressed.

I have read this submission. I believe that I have an appropriate level of expertise to confirm that it is of an acceptable scientific standard.

# Peer review: Referee response for version 1

**DOI:** 10.5256/f1000research.6364.r7782

**Published:** 2015-03-18

**Authors:** Krzysztof Gorgolewski

**Affiliations:** 1 Department of Psychology, Stanford University, Stanford, CA, USA

I wholeheartedly support novel approaches to publishing, especially those run by scientists for scientists and free of commercial interests. Even though I believe a news article would be a better place for PRN to advertise itself, I am happy to review this paper as a "Software Tool Article". I believe addressing the following issues will make the manuscript better:

1. Discussion of "peer review" is very confusing. Putting the term in inverted quotes does not improve readers understanding. In one paragraph you write "As a preprint service, PRN has no peer review process and no editing service." just to follow by "Articles at PRN will be peer viewed by interested readers and also by consultants."
2. There is a lot of future tense used in the paper. This combined with no signs or examples of the platform being used gives the impression that you are describing features you plan to implement rather than existing and mature software solution.
3. It would be good to show how does this service compare to using other existing preprint servers combined with peer review platforms such as Publons or PubPeer.
4. Could anyone review a preprint using your platform?
5. Will the review be public and signed or anonymous?
6. Are the preprints indexed by Google Scholar (in contrast to just Google Web)?
7. I could not find a link to the code of your platform (this is a formal *F1000Research *requirement).

I have read this submission. I believe that I have an appropriate level of expertise to confirm that it is of an acceptable scientific standard, however I have significant reservations, as outlined above.

# Response

**Published:** 2015-08-06

**Authors:** Chaogan Yan

**Affiliations:** Nathan Kline Institute for Psychiatric Research, USA

1. Thank you very much for pointing out this confusion. In the revised manuscript, we have revised all “peer viewing” into “open discussion”.

2. We have revised the future tense to past tense and present tense, as the features have already been implemented.

3. Comparing with arXiv, we have an online commenting system. Comparing with bioRxiv and PeerJ Preprints, we focus on a specific research field (R-fMRI), and invite a panel of consultants to comment (notifications will be sent to all ~5000 R-fMRI members). We are in the process of negotiating with Publons to give credits to the members who give review comments.

4. Yes, we had launched an online review and comment system.

5. Review comments are public and signed. They are intended to help the authors to improve their manuscript(s) for further submission to formal journals. These early feedback would be helpful for authors in revising and improving their articles for later peer review process of traditional scientific journals.

6. We are making efforts to let the preprints to be indexed by Google Scholar. It’s still in progress.

7. The codes have been released through Github (https://github.com/Chaogan-Yan/rfmri.org), we have included this link in the revised manuscript.

# Peer review: Referee response for version 1

**DOI:** 10.5256/f1000research.6364.r7780

**Published:** 2015-03-09

**Authors:** Wei Gao

**Affiliations:** 1 Biomedical Research Imaging Center, University of North Carolina, Chapel Hill, NC, USA

Well done for this initiative! Glad to see this is happening for the community of resting state fMRI.

I have read this submission. I believe that I have an appropriate level of expertise to confirm that it is of an acceptable scientific standard.

# Peer review: Referee response for version 1

**DOI:** 10.5256/f1000research.6364.r7125

**Published:** 2015-01-07

**Authors:** Ze Wang

**Affiliations:** 1 Department of Psychiatry, University of Pennsylvania, Philadelphia, PA, USA

I found it is a great idea to have this kind of service for neuroimaging. One comment that can improve the acceptability of this preprint service is: should the service require authors to notice the normal journals that this manuscript has been archived in PRN? And should PRN share the review comments for the accepted preprints with the standard journals?

I have read this submission. I believe that I have an appropriate level of expertise to confirm that it is of an acceptable scientific standard, however I have significant reservations, as outlined above.

# Response

**Published:** 2015-01-14

**Authors:** Chaogan Yan

**Affiliations:** Nathan Kline Institute for Psychiatric Research, USA

Thank you very much for your comments!

We request the authors to reveal that the papers have been archived in PRN while submitting to preprint compatible journals.

We will share the comments with traditional journals, and encourage the authors to to enclose with their submission.  The PRN is trying to work together with publons ( publons.com) to get credits for the commenters/reviewers.

Thanks,

Chao-Gan and The PRN Team
